# Associations of physical and mental health problems with chronic cough in a representative population cohort

**DOI:** 10.1186/1745-9974-5-10

**Published:** 2009-12-16

**Authors:** Robert J Adams, Sarah L Appleton, David H Wilson, Anne W Taylor, Richard E Ruffin

**Affiliations:** 1 The Health Observatory, Discipline of Medicine, University of Adelaide, The Queen Elizabeth Hospital Campus, Woodville, South Australia, 5011, Australia; 2 Population Research and Outcome Studies Unit, South Australian Department of Health, Adelaide, South Australia, 5000, Australia

## Abstract

**Background:**

Although chronic cough is a common problem in clinical practice, data on the prevalence and characteristics of cough in the general population are scarce. Our aim was to determine the prevalence of chronic cough that is not associated with diagnosed respiratory conditions and examine the impact on health status and psychological health, in a representative adult population cohort

**Methods:**

North West Adelaide Health Study (n stage 1 = 4060, stage 2 = 3160) is a representative population adult cohort. Clinical assessment included spirometry, anthropometry and skin tests. Questionnaires assessed demographics, lifestyle risk factors, quality of life, mental health and respiratory symptoms, doctor diagnosed conditions and medication use.

**Results:**

Of the 3355 people without identified lung disease at baseline, 18.2% reported chronic cough. In multiple logistic regression models, at follow-up, dry chronic cough without sputum production was significantly more common in males (OR 1.5, 95% CI 1.1, 1.9), current smokers (OR 4.9, 95% CI 3.4, 7.2), obesity (OR 1.9, 95% CI 1.3, 2.9), use of ACE inhibitors (OR 1.8, 95% CI 1.1, 2.9), severe mental health disturbance (OR 2.1, 95% CI 1.4, 3.1) and older age (40-59 years OR 1.7 95% CI 1.2, 2.4; ≥ 60 years OR 2.1 95% CI 1.3, 3.5). Among non-smokers only, all cough was significantly more common in men, those with severe mental health disturbance and obesity.

**Conclusions:**

Chronic cough is a major cause of morbidity. Attention to cough is indicated in patients with obesity, psychological symptoms or smokers. Inquiring about cough in those with mental health problems may identify reversible morbidity.

## Background

Cough is the commonest symptom seen in primary care [1-3], and chronic cough is one of the most frequent reasons for new referrals to specialist pulmonologists [4]. However, data on the prevalence of cough lasting more than eight weeks in the general population are scarce [5,6]. Most reports of the prevalence of chronic cough in adults originate from specialist cough clinics and therefore reflect the experience of chronic cough in secondary or tertiary care. The prevalence of chronic cough (lasting more than eight weeks) has been variously reported at 10% to 30% [5,7,8]. Where population data exist they are limited by methodological problems, including use of selected age groups [9-13], self selection of questionnaire respondents [6], failure to differentiate between acute cough due to infection and chronic cough [14]; or a lack of information on other respiratory conditions [10] making it difficult to differentiate the impact of chronic cough from that of airways diseases such as asthma.

Chronic cough is associated with adverse effects on health-related quality of life [15-17]. Successful treatment of cough often leads to major improvement in quality of life [15,16]. Chronic cough is also associated with psychosocial problems that may be more pronounced than physical effects [6,15,16,18]. However, the few studies that have evaluated the impact of cough on health status or psychological health have sampled from specialist clinic populations [19,20] rather than the general population. Others studies are limited by the lack of use of a validated instrument of psychological health [6].

Our aim was to determine the prevalence of chronic cough in a representative adult population cohort, particularly cough that is not associated with diagnosed respiratory conditions, and examine the impact on health status and psychological health.

## Methods

### Sample population and study method

The North West Adelaide Health Study (NWAHS) is a representative biomedical longitudinal population cohort study of people aged eighteen years or older, randomly selected from the electronic white pages telephone directory and living in the north western suburbs of Adelaide, South Australia (regional population 0.6 million). NWAHS initially recruited between 2000 and 2002 with follow-up in 2004-05. The methods of the North West Adelaide Health Study [21] and the validity of these methods of selection to achieve an unbiased sample have been described previously [22]. In particular, there were no major differences between study participants and the comparison population in terms of health indicators or lifestyle behaviours [23].

At stage 1, 4060 adults underwent biomedical examination, representing 69% of those who completed the telephone interview. Overall, at Stage 2 follow-up (mean follow-up time = 3.5 years, range 1.7-5.8 years) survey data was obtained on 88% (n = 3574) and clinic data on 79% (n = 3206) of the Stage 1 NWAHS population using the same methodology and questions. One hundred subjects were deceased, 226 persons were unable to be contacted, and 160 refused further participation in the study.

Telephone interviews investigated self-reported health status (including asthma and COPD), smoking status and demographic variables. A self-completed questionnaire comprised items on demographic information, risk factors (smoking, alcohol use), quality of life, mental health and respiratory symptoms. Smoking was categorized into self-reported current, former or never smoker. Clinic assessment by trained technicians included spirometry according to American Thoracic Society criteria [24], skin prick testing to a panel of eight common allergens, and measurement of height, weight. Obesity was classified as follows: Body mass index [25] (BMI) in kilograms/metre^2^: Underweight: ≤ 18.49; Normal: 18.5-24.9; Overweight: 25.0-29.9; Obesity: ≥ 30.0. Medication use was identified when participants were also asked to bring all current medicines (including complementary medicines) into the clinic at their appointment.

### Respiratory measures

Asthma was defined as current self-reported physician-diagnosed asthma or demonstration of a significant bronchodilator response (SBR) of at least 12% of baseline FEV_1 _in the absence of a doctor diagnosis of asthma [26,27]. Participants with persistent airways obstruction (post-bronchodilator FEV_1_/FVC ratio less than 0.70) [28] were identified. Respiratory symptoms were assessed with the validated Chronic Lung Disease (CLD) Index [29,30]. This is a 6-item instrument that includes items relating to frequency and intensity of dyspnea and wheeze and frequency of coughing and volume of sputum production. Chronic cough was defined as cough reported on most/every day in the past three months. Sputum was defined as at least 2 or 3 tablespoons per day.

### Quality of life and Psychological measures

Health-related quality of life was assessed using the Medical Outcomes Study Short Form 36 Health Survey (SF-36) Physical Health Component Summary (PCS) and Mental Health Component Summary (MCS) scores [31]. The PCS score is constructed such that the mean for the general population is set at 50 with a standard deviation of 10, and higher scores indicate better quality of life [32]. At Stage 1 psychological health was measured by the General Health Questionnaire (GHQ-28), a well-validated and extensively used instrument designed to measure current psychiatric and affective disorders with a focus on disruptions to normal functioning rather than life-long traits [33]. The GHQ-28 contains four subscales: anxiety and insomnia, somatic symptoms (other than cough), social dysfunction, and severe depression [34], providing more information than that of a single severity score [34]. It screens, therefore, for acute rather than chronic conditions [35]. Scores can be interpreted as indicating the severity of psychological disturbance on a continuum [35]. In Australian community populations the GHQ-28 has shown sensitivity of 90% and specificity of 94% for clinically confirmed diagnoses based on the Composite International Diagnostic Interview [36]. At follow-up the GHQ-12 was used, which excludes items most usually selected by physically ill individuals. The GHQ-12 has shown very similar figures to the GHQ-28 in validation studies [35].

### Statistical analysis

Data were weighted to the 1999 Estimated Residential Population for South Australia [37] and Census data [38] by region, age group, gender and probability of selection in the household, to provide population representative estimates. Data were analyzed using the Statistical Package for the Social Sciences (*SPSS Version 15.0*, SPSS Inc, Chicago, IL). Multivariable logistic regression analyses were conducted to assess the association of GHQ disturbance with chronic cough (all, dry, cough with sputum) after adjustment for sex, age, smoking, BMI and ACE inhibitor use. The models were also adjusted for reflux medication use as a proxy for GERD. An additional model assessing the association of all cough and GHQ disturbance was conducted in the population of never/former smokers.

Approval for the NWAHS study was obtained from institutional ethics committees of the North West Adelaide Health Service, and all subjects gave written informed consent.

## Results

The socio-demographic characteristics and health status of the study subjects at Stage 1 baseline have been described in detail previously [21,23]. Of the 3206 people who attended for biomedical assessment at follow-up, doctor-diagnosed current asthma was reported by 439 people (13.5%). Emphysema had been diagnosed in 43 (1.3%) and chronic bronchitis in 239 (7.3%). Airways obstruction (post-bronchodilator FEV1/FVC <70%) was found in 150 (4.8%), and significant acute FEV1 reversibility (>12% & 0.2 L) in 128 (4.1%).

The prevalence of chronic cough at baseline within various demographic and clinical groups is shown in Table 1. Among people without identified airways or restrictive respiratory disease, chronic cough with or without sputum was more common in males, current smokers, those aged less than 40 and over 55 years, and in those with GHQ-28 identified psychological morbidity. Chronic cough was more common across the GHQ-28 domains of anxiety and insomnia, somatic symptoms, social dysfunction and severe depression. Table 2 shows the prevalence of chronic cough by type, in relation to participant characteristics at follow-up. Among people without identified respiratory disease, chronic cough was more common in males, current smokers, participants with high levels of psychological disturbance, and fair to poor general health, and in those using ACE inhibitors. Dry cough, which was more prevalent in older participants, was more common than cough productive of sputum across all population categories, including smokers. The prevalence of cough was not significantly different between former smokers and those who had never smoked.

**Table 1:** Prevalence (%) of all cough within baseline categories of demographic characteristics and mental health status in subjects without identifiable respiratory disease (n = 3355).

		Cough +/- sputum
All subjects (n = 3960)		20.5

No respiratory disease (n = 3355)		18.2

Sex	Male	18.5
	Female	16.0

Smoking status	Current	29.6
	Former	15.6
	Never	11.8

Age (years)	< 40	20.0
	40-54	12.2
	55 and over	18.7

GHQ 28		
Mental health condition	Yes	26.9
	No	14.2

Somatic symptoms	Yes	27.6
	No	14.0

Anxiety and insomnia	Yes	23.6
	No	15.4

Social dysfunction	Yes	28.3
	No	15.2

Severe depression	Yes	34.4
	No	15.7

**Table 2:** Prevalence of dry and productive cough at follow-up in subjects with and without identifiable respiratory disease according to respiratory conditions, demographic characteristics, and health status.

		Dry cough	Cough + sputum
All subjects (n = 3206)		12.1	4.6

Respiratory disease			
Asthma* (433)		25	6
Emphysema^† ^(43)		37	23
Chronic bronchitis^† ^(227)		22	12
Airways obstruction** (150)		30	11
≥ 12% FEV_1 _reversibility (129)		14	9

No respiratory disease (n = 2408)		8.8	3.8

Sex	Male	10.3	4.9
	Female	7.3	2.6

Smoking status	Current	20.9	7.3
	Former	6.6	2.4
	Never	5.5	3.3

Age (years)	< 40	6.3	4.9
	40-54	10.5	3.6
	55 and over	10.4	1.9

Atopy	Yes	9.2	4.3
	No	8.0	2.9

ACE inhibitor use	Yes	14.4	2.7
	No	8.3	3.8

GHQ disturbance	High	15.9	7.8
	Low/none	7.7	3.2

Self-rated health general health	Fair/Poor	19.3	6.0
	Good/excellent	8.8	2.2

SF - 36 Mean (SE)	PCS	44.5 (0.5)	44.7 (0.8)
	MCS	49.6 (0.6)	47.8 (1.0)

* asthma: self reported current doctor diagnosed.

^† ^Self reported doctor diagnosed emphysema and chronic bronchitis,

** Airways obstruction = post-bronchodilator FEV1/FVC < 0.07

In multiple regression analysis (Table 3), chronic cough without sputum production was seen more commonly in males, current smoking and with ageing. There were significant positive associations with severe depression, obesity and use of ACE inhibitors. Modest, but marginally non-significant associations were seen with atopy, and use of anti-reflux treatment. Cough productive of sputum was also more common in males and current smokers, and less common in those who were overweight. Again, a significant association was seen with severe depression. Cough with sputum was not significantly associated with use of ACE inhibitors or anti-reflux treatment. When the analysis was confined to only non-smokers, all cough was more common in men; those with severe depression, the obese, and those aged over 60 years. Again, non-significant associations were seen with atopy, and ACE-inhibitor use (Table 3). When models were analyzed without the GHQ variable, no changes were seen in the size of the associations with other variables and cough.

**Table 3:** Multivariate logistic regression models for cough at follow-up in those without identifiable respiratory disease (n = 2408) and among never/ex-smokers (n = 1938).

	All cough		Dry cough	Cough+sputum
	**All subjects**	**Non-smokers**		

	**OR (95%C.I.)**	**OR (95%C.I.)**	**OR (95%C.I.)**	**OR (95%C.I.)**

**Male**	1.6 (1.3-2.1)	1.5 (1.05-2.0)	1.4 (1.02-1.9)	2.3 (1.4-3.6)

**Age**				
18-39	1.0	1.0	1.0	1.0
40-59	1.5 (1.1-2.0)	1.4 (0.9-2.0)	1.9 (1.3-2.7)	0.7 (0.4-1.2)
60+	1.7 (1.1-2.5)	1.8 (1.2-2.8)	2.6 (1.6-4.1	0.6 (0.3-1.3)

**GHQ disturbance**				
mild-moderate	1.03 (0.7-1.6)	0.9 (0.5-1.6)	1.1 (0.7-1.8)	0.8 (0.4-1.8)
severe	2.2 (1.6-3.0)	1.8 (1.2-2.9)	2.1 (1.4-3.1)	2.6 (1.5-4.3)

**Smoking status**				
former smoker	0.9 (0.7-1.3)	-	1.01 (0.7-1.5)	0.8 (0.4-1.4)
current	4.1 (3.0-5.5)	-	5.4 (3.8-7.8)	2.5 (1.5-4.0)

**ACE inhibitor use**	1.6 (1.02-2.5)	1.4 (0.9-2.3)	1.8 (1.1-2.9)	1.1 (0.4-2.9)

**Atopic**	1.3 (0.97-1.7)	1.3 (0.9-1.8)	1.2 (0.9-1.7)	1.4 (0.9-2.2)

**Anti-reflux agents**	1.3 (0.8-2.0)	1.2 (0.7-2.0)	1.3 (0.8-2.0)	1.5 (0.7-3.4)

**BMI**				
overweight	1.00 (0.7-1.4)	1.2 (0.8-1.8)	1.4 (0.96-2.1)	0.5 (0.3-0.8)
obese	1.5 (1.1-2.0)	1.7 (1.1-2.6)	2.0 (1.2-2.9)	0.9 (0.5-1.5)

Participants reporting cough at any time were significantly more likely to have psychological disturbance on the GHQ-12 and report significantly lower quality of life compared to those without cough at any time (Table 4). Compared to people with cough at both time points, those with cough only at follow-up only had significantly higher mean PCS scores and a lower prevalence of severe psychological disturbance on the GHQ-12, although this was not statistically significant. Compared to people with cough at both time points, those with cough only at baseline had higher mean levels of both PCS and MCS scores, and a lower prevalence of any type of psychological disturbance on the GHQ-12, although this was not statistically significant (p = 0.1).

**Table 4:** Prevalence of GHQ-12 disturbance and SF-36 PCS and MCS scores [mean (SE)] among those with and without cough at baseline and follow-up.

Cough		% GHQ disturbance (n)		SF-36 Mean (SE)	
		**severe**	**≥ mild**	**PCS**	**MCS**

**Baseline**	**Follow-up**				

No	No	10.0 (182)	21.0 (381)	51.4 (0.2)	52.8 (0.2)
	Yes	21.2* (33)	31.4* (49)	47.0* (0.7)	47.2* (0.7)

Yes	No	16.9 (40)	30.9 (73)	47.4 (0.6)	49.0 (0.6)
	Yes	24.8* (35)	36.2* (51)	44.6*†‡ (0.8)	46.3*‡ (0.8)

* p < .01 vs never cough

^† ^p < .01 vs cough only at follow-up

^‡ ^p < .01 vs cough only at baseline

## Discussion

In a representative population sample we have shown that chronic, dry cough is common among people without known respiratory disease, with a prevalence of nearly 9% among adults. Cough productive of sputum occurs in around a further 4% of those without known lung disease. People with chronic cough report significant impairments in quality of life and psychological health, compared to those without cough. Across the population, chronic cough was significantly associated with obesity and severe depression, and was more common in men and in people aged over 60 years. Although cough was more common in people who currently smoke, when only non-smokers were analyzed, the significant associations seen with depression, obesity, men and age persisted. The prevalence of cough was not significantly different between former smokers and those who had never smoked.

The frequency of chronic cough independent of other lung disease, with its strong associations with impaired mental health, particularly depression, and significantly reduced quality of life, indicates cough is a major contributor to morbidity in the community. The reduction in quality of life in general physical health is similar to that previously reported in Australian populations for asthma [39], diabetes [40], arthritis [41] and depression alone [40]. Although use of a cough-specific quality of life instrument may have elicited issues more closely related to cough, the SF-36 correlates well with instruments such as the Leicester Cough Questionnaire [17]. That major impairments were seen in a general health instrument indicates that chronic cough is not a minor problem and deserves thorough evaluation and treatment, particularly as most patients are able to respond to treatment for chronic cough [42].

Our data demonstrates that careful attention should be given to assessment and management of psychological morbidity in the large number of patients with chronic cough in the community, as well as those seen in referral centers. This may be especially the case in people in whom coughing persists in the absence of an identifiable cause and despite extended trials of empirical therapy [43]. Chronic cough was common in smokers and smoking is associated with depression and mental health problems [44]. However, we found the association between chronic cough and disturbance on the GHQ remained strong when only non-smokers were included in the analysis. Under-diagnosis of depression in patients with somatization, particularly major depression, has recently been identified as a significant problem in primary care [45]. Conversely, inquiry regarding cough in patients with mental health problems may also be crucial in identifying reversible morbidity in this group. In one study, successful treatment of cough was correlated with improvements in depression scores in 70% of patients [19].

We found obesity to be significantly associated with dry cough and cough in never/ex-smokers. Janson et al have reported cough was significantly associated with obesity. However, the study population of 20-48 year olds included people with asthma and other respiratory diseases [9]. As obesity has been shown to be significantly associated with asthma, it was unclear from that study whether obesity was linked to chronic cough independently of airways diseases. One possibility is that obesity increases the risk for gastro-esophageal reflux that is contributing to chronic cough in people with obesity. Regardless, our study indicates that chronic cough, with the concomitant problems of impairments in quality of life and mental health, needs to be added to the burden and morbidity of obesity in the community.

Comparison with previous studies examining the prevalence and associations of chronic cough are difficult due to differences in sampling and other methodological questions. We used a validated symptom score of chronic lung disease to identify cough frequency over the previous 3 months. The use of this tool differs from prior studies and makes direct comparison of prevalence between studies difficult. Studies using selected age groups have either excluded adults aged > 50 [9] or > 60 years [11] in whom chronic cough is common [11], thereby missing a large proportion of people with chronic cough. Coultas et al reported a prevalence of cough of 9.3% in people without airflow obstruction from US population data but limited the analysis to adults aged at least 45 years and did not analyse any associations with obesity or psychological disturbance [13]. Zemp et al reported the prevalence of chronic bronchitis symptoms over ≥ 2 years in adults aged less than 60 years [11]. Similar to our data they found no difference in prevalence in cough with sputum between never and former smokers (7%), with cough more common in current smokers (16.7%) [11]. Another community-based study sampled members of the public who requested an information sheet following a national UK radio broadcast, with risk of self-selection of questionnaire respondents [6]. Studies differentiating between infection related acute cough and chronic cough were limited by a lack of information on other respiratory conditions [10] or lung function [46] limiting the ability to differentiate the impact of cough from that of airways diseases such as asthma. Other population studies did not differentiate acute from chronic cough [14]. The strength of our study is that it comes from a representative population sample that was able to identify people with cough over a 3-month period, and those with airways obstruction or restriction on spirometry, previously diagnosed respiratory disease, and current medication use, adding to the generalizability of the findings.

Similar to other reports we found chronic cough is associated with adverse effects on health-related quality of life [15-17] and psychological problems [6,15,16,18]. However, previous studies reporting increased levels of emotional upset have been limited to small numbers of patients referred to specialist cough clinics [19,20]. As only a small part of the population identified in epidemiological surveys seek medical help or advice for cough [5] the population burden of disease from psychological problems associated with cough cannot be extrapolated from these studies. These studies in selected populations have revealed increased levels of depression [19,20] and anxiety using validated questionnaires [20] at frequencies comparable or in excess of that seen in other serious chronic diseases, such as diabetes, asthma or HIV-AIDS [47-49]. Other reports linking cough to psychological morbidity have either not used a validated instrument of psychological health [6] or were unable to specify the frequency, quality, duration, or intensity of reported coughing making it difficult to identify the contribution of chronic cough to this finding [10]. When the GHQ variable was removed from the model the strength of associations with other variables did not change, suggesting the association between psychological disturbance and cough is not acting directly through other factors.

The direction of causality regarding cough and psychological problems is difficult to determine. We found that in terms of disturbance on the GHQ-28 that the group with cough a follow-up only was not significantly different from those with cough at both time points, suggesting there may be little effect of chronicity over our follow-up period of 4 to 5 years. However, we do not know if people had cough for all the follow-up period or recurrent cough only in the 3 months prior to each clinic assessment. Although those people with cough at baseline but who were no longer coughing had significantly higher physical health quality of life and were less likely to report disturbance on the GHQ. This can be interpreted as indicating chronic cough has both immediate and longer-term consequences for psychological health that may stem from the significant impact on general health experienced with cough. Alternatively, this may suggest chronic cough is more likely to be seen in those with underlying anxiety or depression, and this may influence an individual's awareness of symptoms. However, anxiety about underlying serious illness has been identified as a concern for most patients with chronic cough [50]. McGarvey and colleagues found no difference in anxiety trait measures between adults with persistent or idiopathic cough compared with those whose cough was successfully treated [20]. There is not a close association between adverse effects of chronic cough and any specific causes, suggesting the adverse effects are related to the chronic cough itself [18]. Successful treatment of cough can improve depression [19]. Furthermore, the GHQ is an instrument designed to identify "the appearance of new phenomena of a distressing nature [34], rather than lifelong traits. It seems likely that there is a complex interplay between cough and psychological traits and problems that may vary with time.

Contrary to anecdotal observations, and consequent to the representativeness of our sample we found cough to be more common in men and in people aged over 60 years, two groups where there is evidence to suggest there is a tendency to under-report symptoms to clinicians [51]. Older population surveys have reported that cough is commoner in men [52,53], but women are more likely to be seen in specialist cough clinics [4,5]. French et al reported that women with chronic cough are more inclined to present for medical attention than men because of greater HRQL impairments and cough-related psychosocial issues such as embarrassment caused by cough induced stress incontinence [54]. Whether men are less likely to report cough as a symptom to primary care practitioners unless specifically asked remains an open question [55]. However, as indicated earlier, given the prevalence of cough and related physical and mental health problems, there is a case to be made that simple enquiry about coughing may be worthwhile as screening tool for men in general practice, particularly in smokers, the obese, those with a history of allergy or from socially disadvantaged backgrounds. Previous population-based studies have excluded older age groups. The consistent association of chronic cough with advancing age in people without other recognized lung disease seen in our study again suggests that efforts at identifying and managing chronic cough and its related problems in older adults may make a major contribution to reducing morbidity in this burgeoning sector of the population.

Our study is limited by a lack of specific information regarding some of the common causes of chronic cough, such as upper airways syndrome or gastro-esophageal reflux disease [4,5]. However, cough was marginally related to atopy, which itself is closely related to allergic rhinitis, a major cause of post-nasal drip syndrome. Also, it is now appreciated that the postnasal drip syndrome, like GERD, may be clinically silent [56], suggesting that self-report of symptoms may not accurately elicit these problems sufficiently to be confident of any associations in population studies. We were unable to identify people with undiagnosed respiratory disease that did not produce airways obstruction or restriction on spirometry, nor those with undiagnosed cough-variant asthma with normal spirometry. However, many people with cough-variant asthma develop wheezing within 3 years [57], and may have been diagnosed between baseline and follow-up. In addition, the similarity in multivariable models when identified asthma and COPD were included or omitted from the analyses suggests the findings are robust. Our survey was limited to households with telephones, but as 97% of the households in the region have telephones and the demographic characteristics were representative of the population of profile of Adelaide overall [37,38], the extent of any bias is likely to be small. There was also a potential bias from survey non-response, although response rates in our sample were higher than comparable biomedical population studies [58]. The strength of this study is the large representative population sample measurements of other known respiratory problems, and low drop-out rate in follow-up, especially in people over 45 years who are more likely to be at risk for chronic cough.

In summary, chronic cough is a common problem that is significantly associated with reductions in physical and mental health. Investigation and management of chronic cough is therefore an important medical need. Patients with a history of smoking, obesity, allergy, or use of ACE inhibitors should be questioned regarding cough and active clinical care pursued. Careful attention to symptoms of psychological disturbance, including somatic symptoms, and their management may help identify depression and reduce the burden of this problem. Conversely, specifically inquiring about cough in patients with mental health problems may identify reversible physical and psychological morbidity in this group.

## Competing interests

SA, DW and AT have no competing interests to declare. RA and RR have received honoraria and speakers fees from Glaxo-Smith Kline over the last 5 years.

## Authors' contributions

RA took main responsibility for conceiving the analysis and for drafting of the manuscript. SA undertook the analysis and contributed to revision of the manuscript. DW, AT and RR contributed to the conception and conduct of the study, to analytical approaches and methods, and to revising of the manuscript. All authors have read and approved the final version of the manuscript.
